# Improved knee flexion following high-flexion total knee arthroplasty

**DOI:** 10.1186/1749-799X-7-22

**Published:** 2012-06-06

**Authors:** David R Lionberger, Mitchell D Eggers, Kathryn E Brewer, Li Fang

**Affiliations:** 1 Southwest Orthopedic Group, L.L.P., 6560 Fannin Street, Suite 1016, Houston, TX, 77030, USA; 2 Foundation for Southwest Orthopedic Research, 6560 Fannin Street, Suite 1020, Houston, TX, 77030, USA

**Keywords:** Osteoarthritis, Gender-specific high-flexion knee prosthesis, Total knee arthroplasty, Body mass index, Range of motion

## Abstract

**Background:**

The application of new techniques and materials in total knee arthroplasty (TKA) continue to be a primary focus in orthopedic surgery. The primary aim of the present study is to evaluate post TKA total range of motion (ROM) among a group of patients who received a gender specific high-flexion design modification implant compared to a control group of patients who received non-gender specific implants.

**Methods and results:**

The control group was comprised of 39 TKAs that were recruited pre-operatively and received the non-gender specific implant while the study group consisted of 39 TKAs who received gender specific implants. The study group yielded an improvement in mean post-operative ROM of 21° at 12 months, whereas the mean improvement in ROM among the control group was 11°. Thus, the study group had a 10° increased ROM improvement (91%) over the control group (p = 0.00060). In addition, 100% of the subjects with gender specific high-flexion implants achieved greater or equal ROM post-operatively compared to 82% for the control cohort. Lastly, women who exhibited greater pre-operative ROM and lower body mass index (BMI) were found to benefit the most with the gender specific prosthesis.

**Conclusion:**

Our study demonstrates that among subjects with a normal BMI, the gender specific high-flexion knee implant is associated with increased ROM as compared to the non-gender specific non-high-flexion implant designs.

## Background

For the past forty years, advancements in approaches to total knee arthroplasty (TKA) have remained a primary focus in the field of orthopedic surgery. The development of intramedullary and extramedullary cutting instruments and enhanced fixation techniques in the 1970’s led to advancements in prosthesis design and surgical expertise in the 1980’s that have facilitated long-term implant survival [1-3]. Few changes in this evolution; however, have resulted in proven, lasting improvement of performance despite the plethora of claims in the literature of early recovery, functional excellence, and patient satisfaction. Recently, gender specific prosthetics have been developed to address anatomical differences between male and female knees. The main objective of the prosthesis designers is to provide an implant comparable to the human knee in fit and performance with the aim of improving ROM and enabling patients to perform daily living tasks without difficulty.

In efforts to produce a knee prosthetic with optimal fit, researchers have compared current prosthetic dimensions to morphological knee measurements of large patient populations undergoing TKA. A critical study that analyzed 337 knee surgeries for distal femur size, revealed a wide aspect ratio (medial-lateral (M/L) dimension divided by the anterior-posterior (A/P) dimension) variation between the male and female populations [4]. Results from the study indicated that prosthetic manufacturers were skilled at supplying implant sizes that fit the average patient within the population of those undergoing TKA. Specifically, the Zimmer NexGen implant sizes lie just above the best fit (least squares regression) line for combined male and female morphological data. However, these “unisex” implants are particularly inadequate at fitting larger-boned women whose femoral A/P measurements exceed 60 mm. For example, a female with a femoral A/P of 65 mm would be fitted for a NexGen unisex implant with a femoral M/L dimension of 77 mm instead of the best fit value of 67 mm, resulting in 5 mm of overhang on each side. A similar mismatch occurs for other manufactures. For example, Duracon (Stryker Howmedica Ostenics) exhibits an average overhang of 4.9 mm for women and only −0.1 mm for men [4]. Such medial or lateral overhang has been conjectured to result in soft tissue irritation complicated balancing efforts.

For a given femoral A/P size, males have a larger or broader femoral M/L dimension; therefore, traditional femoral prosthetics in women tend to be oversized. In addition, women have less prominent anterior condyles. Poilvache and Insall [5] reported an average lateral anterior condyle thickness in men of 13.7 mm as compared to 12.3 mm in women, while the average medial anterior condyle thickness in men was 10.6 mm in contrast to 9.0 mm in women. The contention is that unisex prosthetics may cause overstuffing of the knee capsule in women that may limit post-operative ROM. Moreover, women have a higher Q angle than men due to their broader pelvic dimension. Several authors [6,7] have established an average Q angle of 14° in men and 17° in women, resulting in a 3° gender difference. These authors suggest that Q-angle variations are linked to the etiology of patellar instability and pain post TKA.

The literature is limited regarding the potential benefits of gender specific knee implants for TKA. The primary aim of the study was to evaluate the performance of a newer TKA design that takes into account the noted high-flexion and anatomical differences in male and female knees and evaluate if those modifications truly make a difference in post-operative ROM.

## Patients and methods

The study protocol was approved by The Methodist Hospital Research Institute in Houston, Texas. A consecutive series of 77 women with a total of 97 TKAs were recruited pre-operatively in an IRB-approved study from the principal investigator’s clinical practice to make up the control group. Each patient underwent primary TKA between November 2005 and October 2006, receiving the Zimmer (Warsaw, Indiana) NexGen CR implant without a high-flexion modification. Of the 77 women, 33 completed their 12-month follow-up visit, yielding 39 total TKAs that were utilized in the analysis. The relatively high rate of attrition can be attributed to loss to follow-up and subjects being excluded if they were unable to comply with the pre-set time points of the post-operative follow-up visits. The study group consisted of 82 women (97 TKAs) who were recruited between October 2006 and February 2008. These subjects received the Zimmer NexGen High-Flex Gender Solutions knee prosthesis. Of the 97 knees, the first 39 TKAs with follow-up data through one year were included in the analysis to yield equal group sizes. A total of 28 of these gender specific implants were NexGen CR-Flex, while the remaining 11 were NexGen LPS-Flex. Additional file 1 provides a sample size calculation for the continuous response variables. All patients of the surgeon for whom TKA was recommended, met all of the inclusion criteria, and met none of the exclusion criteria, were offered participation in the study. Subjects were not exposed to advertising of any specific type of implant at the hand of the investigator. While there were variations in design and cruciate stabilization in the study cohort, we segregated each subset to verify no differences in CR versus LPS exhibited before merging the group into one study group.

All surgeries were performed by the principal investigator utilizing computer-assisted navigation via a minimally invasive (quad sparing) technique with the same gap settings and extension passive ranges in order to normalize surgical technique mismatch. This approach ensured that early patients did not have an advantage due to differences in range under anesthesia or restrictive tightness on ligament tensioning. The control and study cohorts received identical pre- and post-operative care.

Exclusion criteria for participation included: under 20 years of age, cancer, major anatomical compromise, and bone deformity or contradiction. The two cohorts revealed no statistically-significant differences in any demographic feature. The mean age and BMI for the control cohort was 68.3 years and 30.0 kg/m^2^ respectively, while the mean age and BMI for the study cohort was 67.9 years and 29.9 kg/m^2^. Also, pre-operative extension and flexion did not prove statistically significant, thereby minimizing the possibility of confounding variables in this study.

Patients were evaluated at three designated post-operative time intervals: 2 months, 6 months and 12 months. All patients within the balanced cohorts of 39 TKAs returned for their first and last post-operative visits. At each post-operative visit, the principal investigator determined extension and flexion using a goniometer. Post-operative complications and all occurrences of post-operative manipulation under anesthesia or implant revision were recorded.

Pre- and post-operative extension, flexion, and ROM and net change (improvement) in ROM between pre-operative and each post-operative visit were analyzed using Welch’s *t*-test with α = 0.05 (twin-tailed) to accommodate unequal variance and sample size. The correlation between post-operative ROM and continuous variables (e.g., pre-operative scores, age, BMI, femoral size) was determined using scatter plots and least squares best-fit trend lines. Statistical differences in the occurrences of complications, manipulation under anesthesia, and revisions for each group were determined using Pearson’s chi-squared test.

## Results

Summary statistics are shown in Table 1 for both control (unisex implant) and study (gender specific implant) subjects. The study group showed no significant difference between CR and LPS design differences when compared to one another. This group of 39 TKA then represented the study cohort of gender high-flexion modification which would serve as the comparison group to the older design NexGen control group. There was no statistically significant difference in mean pre-operative ROM found between the study and control groups; 102.7° and 107.2° respectively. At 2 months, the ROM for the study group improved by reaching an average 10° improvement from baseline, whereas the control group reached an average 2° improvement, which was statistically significant (p < 0.05). At 6 months, the control group reached a mean ROM of 115° (10.8) compared to 119.2° (10.2) among the study group, representing equal improvement from the 2-month follow-up visit, but a 16.3° improvement from baseline for the study group and a 6.5° improvement from baseline for the control group. At the final 12-month follow-up visit, the control group averaged a 10.8° improvement over baseline as compared to 20.7° among the study group. When separating the control group where high-flexion without gender was used, there was not a difference.

**Table 1:** Summary statistics

	**Pre-Op**		**2 Month Post-Op**		**2 Month ∆**		**6 Month Post-Op**		**6 Month ∆**		**12 Month Post-Op**		**12 Month ∆**	
	**mean**	**std dev**	**mean**	**std dev**	**mean**	**std dev**	**mean**	**std dev**	**mean**	**std dev**	**mean**	**std dev**	**mean**	**std dev**
**Gender Knee**														
N	39.0		39.0		39.0		35.0		35.0		39.0		39.0	
extension	10.8	4.0	4.0	3.4	(6.8)	4.6	1.7	2.1	(9.0)	4.0	0.7	1.4	(10.1)	3.9
flexion	113.5	8.7	117.1	9.3	3.5	11.0	120.9	9.7	7.3	11.1	124.1	9.5	10.5	10.3
ROM	102.7	9.5	113.1	11.0	10.3	12.7	119.2	10.5	16.3	11.9	123.4	9.8	20.7	11.0
**Control Knee**														
N	39.0		39.0		39.0		30.0		30.0		39.0		39.0	
extension	9.5	4.6	4.6	3.9	(4.9)	5.4	2.6	3.5	(7.0)	5.7	1.3	2.6	(8.2)	4.8
flexion	117.0	9.8	113.8	8.1	(3.2)	11.3	117.6	8.4	(0.5)	10.6	119.6	8.4	2.6	11.2
ROM	107.5	11.5	109.3	10.6	1.7	13.4	115.0	10.8	6.5	13.8	118.3	9.7	10.8	13.2
**Difference (G-C)**		p-value		p-value		p-value		p-value		p-value		p-value		p-value
extension	1.3	0.18779	(0.5)	0.51676	(1.8)	0.10828	(0.9)	0.25079	(2.0)	0.11190	(0.6)	0.18911	(1.9)	0.05408
flexion	(3.5)	0.09932	3.2	0.10605	6.7	0.00949	3.3	0.14166	7.8	0.00530	4.5	0.03096	7.9	0.001649
ROM	(4.8)	0.04836	3.8	0.12750	8.6	0.00485	4.2	0.11872	9.8	0.00353	5.1	0.02317	9.9	0.000595

**Figure 1:**
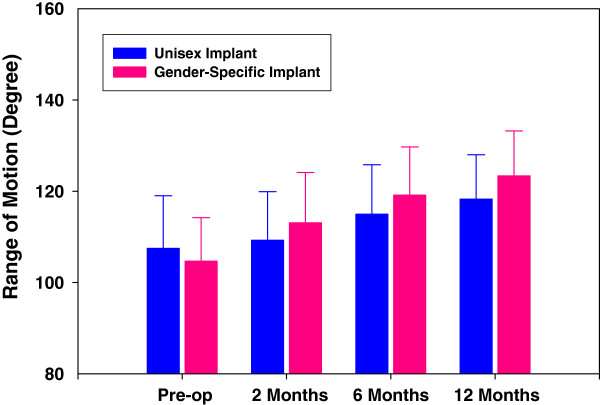
ROM vs. Visit.

**Figure 2:**
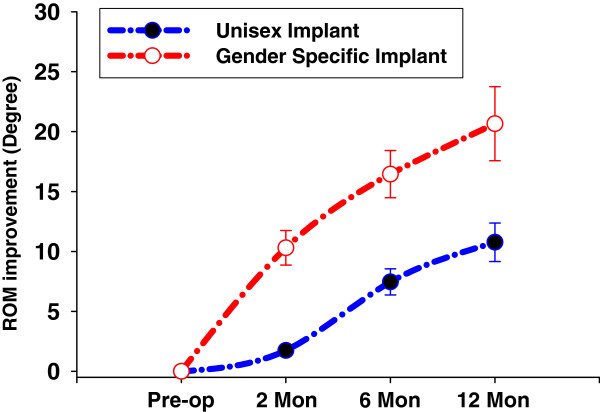
ROM Improvement vs. Visit.

The progression of ROM improvement from pre-operative values for both cohorts is illustrated in Figure 1. The least square logarithmic trend line was adopted to reveal the asymptotic progression to the noted 10° differential between gender specific high-flexion designs versus the unisex implant group. At the 12-month evaluation, mean ROM improved for both implants-the gender specific prosthesis improved ROM by approximately 21° compared to 11° for the control group representing a 91% improvement (refer Figure 2). 

**Figure 3:**
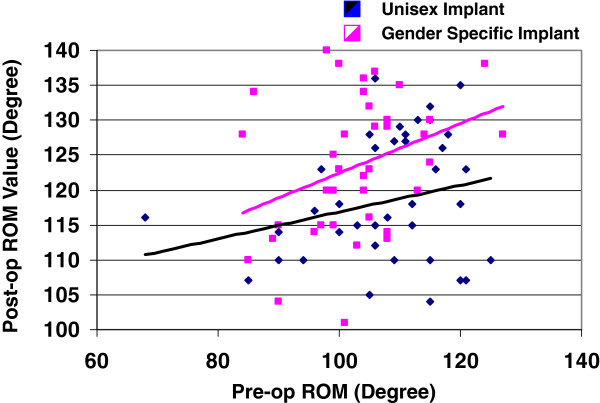
Post-Op ROM vs. Pre-op ROM.

The post-operative (12 months) was analyzed with respect to pre-operative ROM for both cohorts to reveal the class of subjects for which greatest post-operative ROM was achieved. Figure 3 provides further illustration of the resulting post-operative ROM versus pre-operative ROM. As expected, the subjects with greatest pre-operative ROM achieved the greatest post-operative ROM as indicated by the linear least squares trend line. Interestingly, the mean (10° differential) was achieved between the cohorts primarily with the higher pre-operative ROM, often corresponding with lower BMI.

Also, as indicated in Figure 3, the best fit lines (minimum least squared error) further reinforces the consistent improvement arising from the gender specific high-flexion design prosthesis compared to the non-high-flexion whether it is for the unisex or gender-based prosthesis. Although the mean improvement using the gender specific implant over the unisex implant was established to be 10°, the figure depicts the improvement actually varying from approximately 4° for subjects with poor pre-operative ROM (90°) to 10° for subjects with high pre-operative ROM (near 120°). Furthermore, all subjects with gender specific implants achieved post-operative ROM at 12 months at least equivalent to their pre-operative ROM. In contrast, 82% of the women with unisex implants realized equal or better ROM at 12 months. Consequently, those women with greater pre-operative ROM benefited most with the gender specific implant relative to the unisex prosthesis, while the more impaired women with low pre-operative ROM experienced, on average, the least ROM improvement irrespective of implant choice.

**Figure 4:**
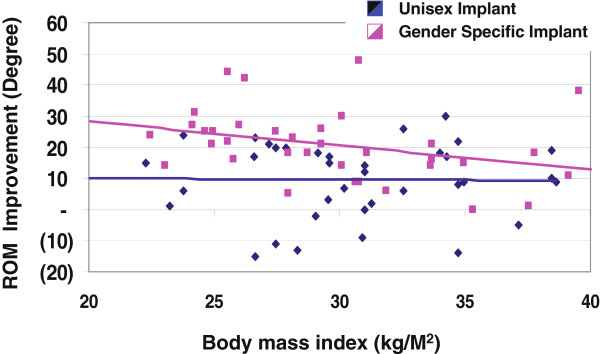
ROM Improvement vs. BMI.

The ROM improvement was further analyzed with regard to BMI for each cohort as illustrated in Figure 4. Improvement for the unisex implants was sporadic, although the linear regression revealed a near constant improvement of 10°. In contrast, the ROM improvement afforded by the gender specific implant revealed a dependency on BMI; namely the more fit the subject, the better the realized ROM gain. As evidenced by the linear regression, for each unit of BMI decrease in the range of 22 to 39 kg/m^2^, ROM improvement increased by 0.8° for the gender specific implant. Based on this analysis, women in the normal range (BMI approximately 20–26) can expect the full gain of the gender specific implant, while obese women (BMI approximately 30–40 kg/m^2^) will likely achieve less than 10° improvement over the unisex implant.

**Figure 5:**
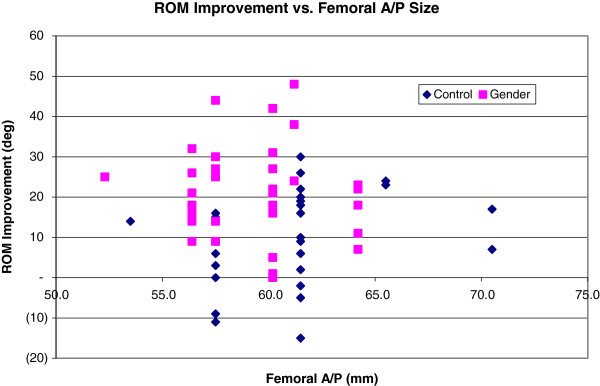
ROM Improvement vs. Femoral A/P Size.

The ROM improvement was also analyzed with respect to femoral implant A/P size to assess if physical size could play a role in improving range when using more modern designs in condylar dimensions. Figure 5 depicts the ROM improvement as a function of femoral implant A/P size for both patient cohorts. For the larger aspect ratio-matched gender specific prosthesis, mean ROM improvement was 18° at femoral A/P = 60 mm (was 60.2 mm x 64.7 mm gender specific implant is matched to 60 mm x 65 mm average distal femoral morphological size as opposed to using the nearest 61.5 mm x 72.0 mm unisex implant), whereas in the size-mismatched unisex implant, the ROM improvement was just 11° at femoral A/P = 62. Such trend supports the hypothesis that appropriately matched femoral implants improve ROM outcome. However, the improvement does not progress beyond femoral A/P = 64. Given the low sample number at such femoral A/P extremes, the gains realized by correct aspect ratio matching is not powered to be significant but does represent an interesting trend where body habitus may not be complimentary in larger patients to present implants.

Finally, a total of 184 knee surgeries were retrospectively analyzed with regard to occurrences of post-operative complications and revisions to compare with the same time frame of one year in the study group. With the exception of revisions, no relationship existed between complications (structural, fibrosis, neurological, and manipulation under anesthesia) and type of prosthetic. However, revisions were 5 times more likely (p = 0.00015, Pearson’s Chi-square test) with the unisex implant than the gender specific implant. The revisions consisted of 4 mechanical failures and one infection.

## Discussion

This study provides evidence to support the notion that newer designs of dimensional matching by aspect ratio and high-flexion modifications may yield early recovery advantages over conventional unisex designs of the past. Specifically, upon examining 78 TKAs, the 39 gender-specific high-flexion knee prosthetics improved pre-operative ROM an average 21° at 12 months, while the 39 conventional designed implants improved ROM by 11°, resulting in a 10° ROM improvement differential (or equivalently, 91%) attributable to the gender-specific implant (p = 0.00060). Computer-assisted surgery was employed in an attempt to hold surgeon related variables constant throughout the study minimizing surgeon bias. Moreover, all subjects with gender-specific high-flexion implants achieved post-operative ROM at least equivalent to their pre-operative value, whereas 82% of subjects with the unisex implant reached or exceeded their pre-operative ROM. Revisions were 5 times more likely (p = 0.00015) with the unisex implant.

Regardless of the choice of implant, subjects with greater pre-operative ROM posted the greatest postoperative ROM. However, those women who exhibited greater pre-operative ROM and lower BMI benefited the most with the gender specific prosthesis as compared to the unisex implant. Specifically, women in the normal BMI range (20–26 kg/m^2^) achieved the full benefit of the gender specific implant, whereas obese women (30–40 kg/m^2^) achieved on average less than the 10° ROM gain possible with respect to unisex implants. As a coarse approximation, for each unit of decreasing BMI, an additional degree of ROM improvement can be expected with the gender specific implant. These BMI-associated results reinforce the notion that non-obese patients fare better in TKA outcome than obese patients as reported by others [8].

A short-range trend, namely the considerably greater gender specific ROM improvement over unisex implants for femoral A/P sizes 60-64 mm, was discovered to support the hypothesis that appropriately matched femoral implants improve ROM outcome. However, given the low sample size for subjects with femoral A/P sizes greater than 62 mm, additional subjects with larger femurs will need to be examined to yield a definitive conclusion. This exhibits one of two limitations and shortcomings of this study.

First, the trend of larger sized condyles not yielding as much ROM in this small subset does raise questions about body habitus, flexibility, and design matching. While the smaller, thinner individuals represent the more typical athletic group, it may be that their perceived improved performance is one of fitness rather than deficiencies of implant designs. The second limitation is the mix of CR and LPS in the study group. While this may represent a potential of confounding variables, there was no apparent difference in magnitude of ROM in the two subsets. This is supported by the paucity of literature articles showing no differences in ROM on functionality in cruciate sparing and sacrificing designs. As both cohorts yielded a 12-month post-operative ROM of at least 118°, both designs can be considered capable of producing a successful outcome with regard to functionality required for the average American lifestyle. However, the high-flexion design may afford the extra flexion required for more athletic subjects and better accommodates lifestyles involving squatting, leg crossing and deep kneeling. Within this subset of higher demand patients, this subtle improvement in ROM may provide the necessary difference between satisfaction or frustration in certain activities.

There were no significant adverse events attributed to either group. Curiously, the historical group used in review of non-gender revision rates for the same time period of follow-up showed a 5 times higher rate of revision than the study group over the 12-month follow-up period. While this was significant, there did not appear to be a single course effect in the historical group solved by the study group. The 5 patients out of 189 reviewed to compare the authors’ historical rate did, however, seem to be improved by the subsequent 39 study patients in the study series. While design may make a difference in failure, this study cannot draw conclusions from such short follow-up or small population.

Collectively, the substantive anatomical differences in male and female knees would be expected to result in different functional TKA outcomes when employing unisex prostheses as we have found. Most studies, however, do not reveal a significant gender difference or bias in clinical outcomes. To further elaborate, Ritter and Eizember et al. [9] found no significant difference in ROM outcome based on gender, but did show significance based on age. In another study, Ritter and Wing et al. [10] evaluated 7326 knees with respect to Knee Society knee score, flexion, pain relief and walking improvement following TKA. They concluded that with regard to clinical outcome measured by these metrics, women perform just as well as men with the unisex prosthesis system. Finally, MacDonald and Charron et al. [11] also could not identify a gender bias in clinical outcome measured by WOMAC, SF-12, and KSCRS. Although females slightly outperformed males in WOMAC and SF-12 improvement following TKA, men slightly outperformed women in KSCRS improvement. This was also noted in our series (larger implants ≥ 64 mm) by the trend in ROM improvement which also did not translate to better motion.

Absence of gender bias in clinical outcome following the use of unisex knee prosthetics has led some to conclude that high-flexion prosthetics are not warranted. However, uniform outcome across gender with unisex prosthetics may support alternative conclusions. First, the unisex implants may be underperforming in men and women in that the average ROM following TKA reported is only 100°-110° [12-14], while the human knee is capable of 160° [15]. In fact, the American Academy of Orthopaedic Surgeons claims that the normal human knee has a passive ROM of 144° and that TKA “success” should be characterized by post-operative ROM greater than 110°. With a better, more personalized prosthetic fit, the mean post-operative ROM may reach well beyond 100°-110°. Secondly, the perceived absence of gender bias may be due to the lack of sufficient resolution in commonly used clinical outcome metrics. A gender bias may be present, yet undetectable with conventional metrics such as WOMAC, SF-12, SF-36, and the Knee Society knee score. For example, the Knee Society knee score developed by Insall [16] discounts ROM to 20% of full value (divides ROM by 5) in comprising the overall score. A substantive 10% change in ROM, for example, from 100° to 110°, accounts for a mere 2% change in KS knee score. Together with a reported intraobserver error of 11% and interobserver error of 16% [17], the ability to detect gender bias becomes challenging when using knee score alone. Finally, it is worth pondering whether the 10° differential in ROM improvement between the gender specific high-flexion implant and the unisex implant is due to the high-flexion nature of the gender specific design, the high-flexion, or both. High-flexion knee prostheses have been designed to achieve flexion well beyond the average 100°-110° ROM by removing an additional 2 mm of bone from the posterior femoral condyle, increasing the articulation curvature during high-flexion activities. In many models, the tibial insert is modified with an anterior cut to avoid patellar tendon impingement during high-flexion. Additionally, NexGen LPS Flex exhibits a modified cam/post to avoid dislocation at the high-flexion to provide a theoretical 150° of flexion.

Unfortunately, the literature reporting high-flexion prostheses is inconsistent in their clinical outcome. Both Laskin [18] and Huang and Su et al. [19] investigated 80 and 50 TKAs respectively and found 14° flexion improvement with high flex implants compared to traditional implants. Also, Weeden and Schmidt et al. [20] and Bin and Nam et al. [21] surveyed 50 and 180 TKAs and reported flexion gains of 12° and 6° respectively. However, Suggs and Kwon et al. [22], Kim and Sohn et al. [23], and Seon and Song et al. [24] found no significant difference in clinical outcome between high-flexion and traditional implants.

Consequently, it remains difficult to assert whether the observed 10° gain in ROM improvement is attributable to the gender-specific characteristics, the high-flex modifications, or a combination of both features of this prosthesis design. A future third cohort utilizing a high flex unisex implant may well resolve such question. Clearly, the combination of high-flexion with or without gender specific appears to be consistent with enhanced ROM.

Overall, advances in knee prosthetic design and TKA surgical techniques have yielded implantable knees with ever increasing comparability to the human knee in fit and performance. Our study demonstrated that the short-term (12 months) ROM improvement of a gender-matched high-flexion designknee prosthetic was 10° (or 91%) superior to the conventional unisex prosthesis. Also, women who exhibited greater pre-operative ROM and lower BMI were found to benefit the most with the gender-specific prosthesis. For each decreasing unit of BMI, an additional degree of ROM improvement can be expected with the gender-specific implant. These modest improvements suggest the optimal knee of the future may well be a *personalized* implant designed uniquely, and manufactured rapidly, for each patient. This study also supports the claim that certain design modifications in the newer implants may aid in producing better functional outcomes; therefore, the orthopedic community should strive to embellish these new developments.

## Competing interests

David R. Lionberger, MD had competing interests as he had consultancies with the following companies: Pfizer, Zimmer, and Proctor & Gamble. All other authors declare that they have no competing interests.

## Authors’ contributions

DRL: data generation, manuscript preparation. MDE: manuscript preparation, statistical analysis and results. KB: data collection. LF: manuscript revision. All authors read and approved the final manuscript.

## Supplementary Material

Additional file 1 Sample size calculations for continuous response variables. Click here for file
