# Supplementary material for: Improved knee flexion following high-flexion total knee arthroplasty
Source: J Orthop Surg Res. 2012 Jun 6;7:22. doi: 10.1186/1749-799X-7-22 (PMC3478993; doi:10.1186/1749-799X-7-22)
Supplement: Additional file 1 — Sample size calculations for continuous response variables. [file 1749-799X-7-22-S1.rtf]

Table 2.

Sample Size Calculations for Continuous Response Variables
a	Side	Power	s	d	Size (2N)	
0.050	2	0.800	8.000	3.000	224	
0.050	2	0.800	8.000	5.000	82	
0.050	2	0.800	8.000	10.000	22	
